# A Pilot Study of Medical Student Opinions on Large Language Models

**DOI:** 10.7759/cureus.71946

**Published:** 2024-10-20

**Authors:** Alan Y Xu, Vincent S Piranio, Skye Speakman, Chelsea D Rosen, Sally Lu, Chris Lamprecht, Robert E Medina, Maisha Corrielus, Ian T Griffin, Corinne E Chatham, Nicolas J Abchee, Daniel Stribling, Phuong B Huynh, Heather Harrell, Benjamin Shickel, Meghan Brennan

**Affiliations:** 1 Department of Medicine, University of Florida College of Medicine, Gainesville, USA; 2 Department of Anesthesiology, University of Florida College of Medicine, Gainesville, USA

**Keywords:** artificial intelligence, chatgpt, large language model, medical education, survey

## Abstract

Introduction

Artificial intelligence (AI) has long garnered significant interest in the medical field. Large language models (LLMs) have popularized the use of AI for the public through chatbots such as ChatGPT and have become an easily accessible and recognizable medical resource for medical students. Here, we investigate how medical students are currently utilizing LLM-based tools throughout medical education and examine medical student perception of these tools.

Methods

A cross-sectional survey was administered to current medical students at the University of Florida College of Medicine (UFCOM) in January 2024 discussing the utilization of AI and LLM tools and perspectives on the current and future role of AI in medicine.

Results

All 102 respondents reported having heard of LLM-based chatbots such as ChatGPT, Bard, Bing Chat, and Claude. Sixty-nine percent (69%; 70/102) of respondents reported having used them for medical-related purposes at least once a month. Seventy-seven point one percent (77.1%; 54/70) reported the information provided by them to be very accurate or somewhat accurate, and 80% (55/70) reported that they were likely to continue using them in their future medical practice. Those with some baseline understanding of and exposure to AI were 3.26 (p=0.020) and 4.30 (p=0.002) times more likely to have used an LLM-based chatbot, respectively, and 5.06 (p=0.021) and 3.38 (p=0.039) times more likely to cross-check information obtained from them, respectively, compared to those with little to no baseline understanding or exposure. Furthermore, those with some exposure to AI in medical school were 2.70 (p=0.039) and 4.61 (p=0.0004) times more likely to trust AI with clinical decision-making currently and in the next 5 years, respectively, than those with little to no exposure. Those who had used an LLM-based chatbot were 4.31 (p=0.019) times more likely to trust AI with clinical decision-making currently compared to those who had not used one.

Conclusion

LLM-based chatbots, such as ChatGPT, are not only making their way into the medical student repertoire of study resources but are also being utilized in the setting of patient care and research. Medical students who participated in the survey generally had a positive perception of LLM-based chatbots and reported they were likely to continue using them in the future. Previous AI knowledge and exposure correlated with more conscientious use of these tools such as cross-checking information. Combined with our finding that all respondents believed AI should be taught in the medical curriculum, our study highlights a key opportunity in medical education to acclimate medical students to AI now.

## Introduction

The use of artificial intelligence (AI) has garnered significant interest in the medical field due to its potential to enhance the speed and accuracy of medical diagnoses, automate administrative tasks, and augment telehealth. One particular type of AI that has become a popular topic of late is large language models (LLMs). A prominent example of this is ChatGPT, an LLM-based chatbot that was launched by OpenAI (San Francisco, California, US) in November 2022. ChatGPT sparked great interest in the medical education community when a study published in PLoS Digital Health found that ChatGPT could pass the United States Medical Licensing Examination (USMLE) Step exams without prior training or reinforcement [1].

Recently developed chatbots, such as ChatGPT, use LLMs to interact with users based on their input prompts. These LLM-powered chatbots generate responses based on complex pattern recognition and statistical synthesis of massive datasets of articles, books, websites, and other written text [2]. In the educational setting, LLMs are being used by students and educators, and their use is expected to increase rapidly [3]. The rapid, and potentially detailed, responses generated by these applications have the potential to revolutionize the way medical students learn and study medicine. Rather than thumb through a textbook or scour the internet for clarification on a topic, students can “ask” ChatGPT to receive a detailed explanation in seconds.

Previous research has examined the applications of LLM-based tools in medical education outside of the United States. These tools excelled in augmenting academic writing, generating practice questions with detailed answers, creating patient-physician simulations, and suggesting new ideas for research [4]. Research by Cross et al. revealed that one-third of full-time faculty at a Caribbean medical school were using ChatGPT [5]. The most common use was to generate multiple-choice questions, and most faculty believed that ChatGPT should be available to students [5]. In addition, cross-sectional studies conducted at multiple international medical institutions found that medical students were currently using LLM-based chatbots and had a favorable attitude toward them and AI in general [6-8]. Cross-sectional studies have also been performed on medical students' opinions of AI in general, which showed that the majority of students believed AI training would be useful in their future careers and that it should be taught in medical education [9-11].

There is still an inadequate understanding of how medical students incorporate LLM-based chatbots, such as ChatGPT, into their education, especially in the United States. We aimed to establish a preliminary understanding of how medical students utilize these tools throughout their education while also examining their perception of them. We conducted a pilot survey of medical students currently enrolled at the University of Florida College of Medicine (UF COM) to assess our students' use of these tools. Identifying the nuanced applications of LLM-based chatbots among medical students will help medical educators optimize these tools for educational purposes and suggest effective uses to their students. We also aimed to use the results of our pilot study to further refine the survey, ultimately seeking to distribute it to medical students on a national level. Presently, there is no national standard for AI education in medical school curricula. However, ongoing discussions focus on how AI tools like LLM-based chatbots could enhance medical education and how best to teach future physicians how to incorporate AI tools into their practice. A broader understanding of medical students’ attitudes and the current use of AI tools, such as LLM-based chatbots, will help us predict how future physicians may incorporate AI into their practice and guide the incorporation and development of an AI curriculum in medical training.

## Materials and methods

Data collection

We designed a cross-sectional survey to capture participants’ experiences and perceptions regarding LLMs with a focus on the current and future use of LLMs in medical education. The survey consisted of 20 questions divided into 4 main sections: demographics, AI baseline proficiency and usage, perception of LLMs, and implications of AI in medical education and healthcare.

While formal validation methods were not employed, the questionnaire was developed in consultation with experts in medical education and artificial intelligence and piloted among a sample of eight medical students to refine the questionnaire who were not included in the survey study.

An IRB-exempt (ET00022207) cross-sectional Qualtrics online survey was distributed to medical students in all years of training at the UF COM in January 2024 via email with weekly reminder emails over the course of four weeks. Participation in this study was voluntary, and no financial compensation or other incentives were provided to participants. 

Data analysis

Statistical analyses were conducted using JMP software (JMP®, Version 15, SAS Institute Inc., Cary, NC, 1989-2023), with the significance level set at p-value < 0.05. Pearson’s chi-square test was used to determine associations between survey responses. Participant responses were categorized into binary groups for analysis purposes due to sample size.

Independent variables included preferred medical residency, stage of medical school (clinical or pre-clinical), prior AI knowledge, and exposure to AI. Responses to these questions were each re-coded as described below into two categories for a 2x2 analysis.

Preferred Medical Residency

Specialties were re-coded as procedural or non-procedural. Procedural specialties included general surgery, anesthesiology, interventional radiology, neurological surgery, obstetrics and gynecology (OBGYN), orthopedic surgery, otolaryngology (ENT), plastic surgery, thoracic surgery, and urology. Non-procedural specialties included all other specialties. Participants uncertain about their preferred medical residency were excluded from analyses involving this variable.

Stage of Medical School

First and second-year medical students were coded as pre-clinical while third and fourth-year students were coded as clinical.

Baseline AI Knowledge

Those who chose “limited or no understanding” were categorized as “limited to no understanding” while those who chose “basic understanding,” “moderate understanding,” “proficient understanding,” or “advanced understanding” were re-coded as "moderate understanding and above.”

Exposure to AI

Those who chose “limited or no exposure” were categorized as “limited to no exposure” while those who chose “moderate exposure,” “some exposure,” “substantial exposure,” or “extensive exposure” were re-coded as “some exposure.”

Dependent variables were also categorized. 

Frequency of Cross-Checking Information

Responses were grouped into two categories: cross-checks infrequently (“never” or “occasionally” cross-checks) and cross-checks frequently (“sometimes,” “often,” and “always” cross-checks).

Likert Scale Responses

Responses of “disagree”, “strongly disagree”, or “neutral” were grouped as “neutral or disagree” while “agree” and “strongly agree” were grouped as “agree.” Responses marked as “N/A” were excluded from the statistical analysis.

Participants who reported never using LLMs, either generally or for medical-related purposes, were excluded from the analysis of specific questions related to LLM usage or experience. Only fully completed surveys were analyzed.

## Results

The survey was sent via an email listserv of 662 medical students at the UF COM in all years of training. One hundred two (102) students completed survey forms. The response rate for this survey was 15.4% (102/622). Most respondents were between the ages of 21 through 29 (Table 1). Seventy-five point five percent (75.5%; 77/102) of respondents were in their pre-clinical years of training (Table 1). Thirty point four percent (30.4%; 31/102) of respondents were interested in a procedural specialty, 57.8% (59/102) were interested in a non-procedural specialty, and 11.8% (12/102) were unsure (Table 1). Seventy-eight point four percent (78.4%; 80/102) of respondents reported having some current baseline understanding or more of AI. Specifically, 43.1% (44/102) reported basic understanding, 23.5% (24/102) reported moderate understanding, 10.8% (11/102) reported proficient understanding, and 1% (1/102) reported advanced understanding. Sixty-five point seven (65.7%; 67/102) reported having some exposure or more to AI in their medical school curriculum, extracurriculars, or research (Table 1).

**Table 1:** Survey respondents' demographics and baseline AI knowledge and exposure n = number. Data have been represented as n, %.

Demographics		Medical students, n (%)
Age (years)		
	Under 21	0 (0.0%)
	21 through 23	38 (37.2%)
	24 through 26	50 (49.0%)
	27 through 29	12 (11.8%)
	Over 29	2 (2.0%)
Year of medical training		
	Preclinical	77 (75.5%)
	Clinical	25 (24.5%)
Interested residency		
	Procedural	31 (30.4%)
	Non-procedural	59 (57.8%)
	Unsure	12 (11.8%)
Current understanding of AI		
	Limited or no understanding	22 (21.6%)
	Basic understanding or more	80 (78.4%)
Exposure to AI in medical school curriculum, extracurriculars, or research		
	Limited or no exposure	35 (34.3%)
	Some exposure or more	67 (65.7%)

**Figure 1:**
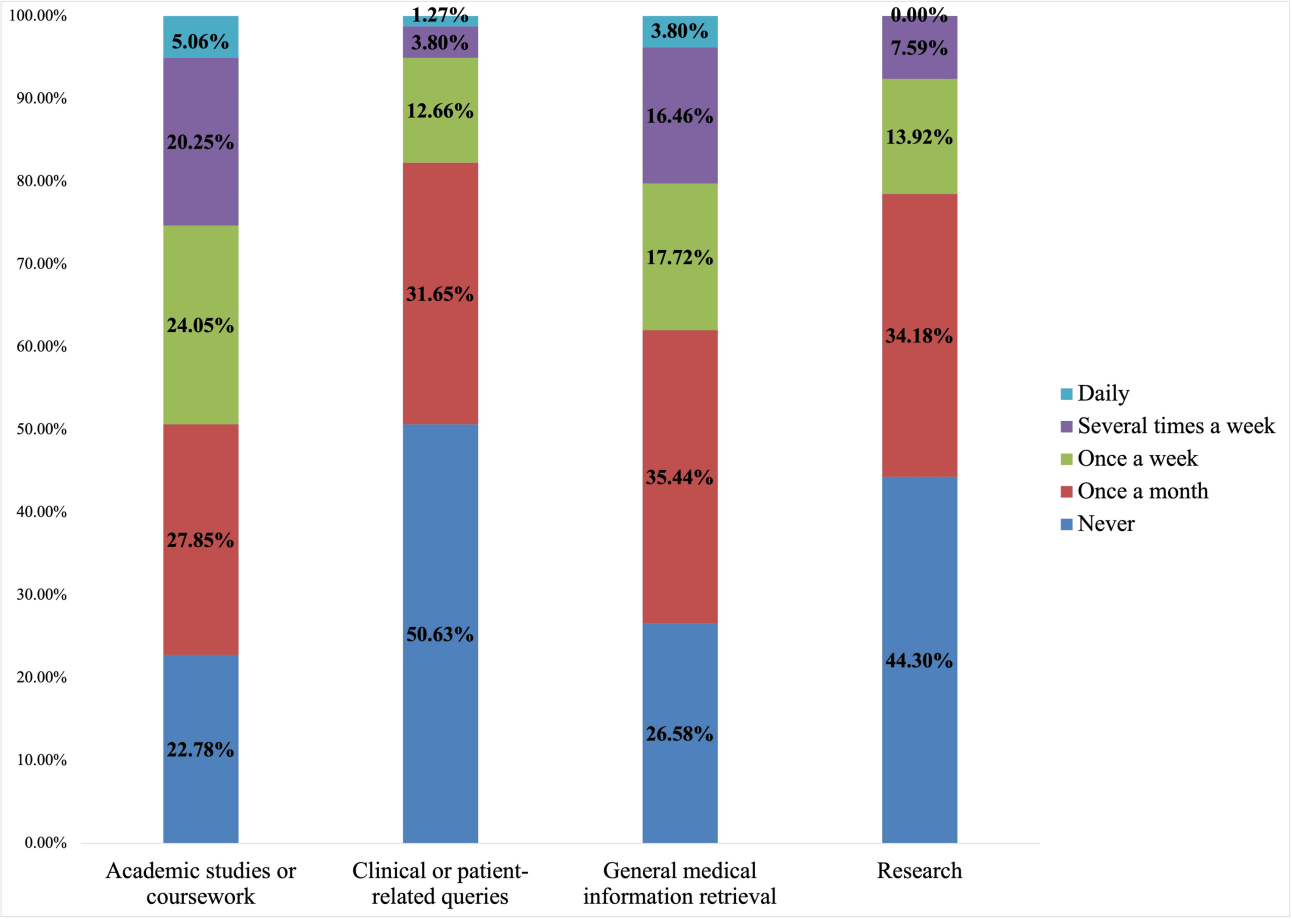
Approximate frequency of use of LLMs for various medical-related purposes of survey respondents The y-axis indicates the proportion of all respondents. LLM: large language model

All 102 respondents reported an awareness of LLMs. Seventy-seven percent (77%; 79/102) of students reported having used an LLM and 69% (70/102) of students reported having used an LLM for medical-related purposes at least once a month. Users of LLMs were asked for the specific breakdown of LLM usage for medical-related purposes, with 56% (44/79) reporting using it for research, 49% (39/79) for clinical or patient-related queries, 77% (61/79) for academic studies or coursework, and 73% (58/79) for general medical information retrieval at least once a month (Figure 1).

Of those who reported using LLMs for medical-related purposes, 77.1% (54/70) reported the information provided by LLMs to be very accurate or somewhat accurate. Eighty percent (80%; 55/70) reported that they were likely to continue using LLMs in their future medical practice. When asked how often they cross-checked information obtained from LLMs, 18.6% (13/70) responded they never cross-checked information obtained from LLMs (Table 2).

**Table 2:** Medical students' perceptions of LLMs n = number. Data have been represented as n, %. LLM: large language model

Questions		Medical students, n (%)
How would you rate the accuracy of information provided by LLMs in addressing medical-related questions or problems?		
	Somewhat inaccurate or very inaccurate	6 (8.6%)
	Neutral	7 (10.0%)
	Somewhat accurate or very accurate	54 (77.1%)
	N/A	3 (4.3%)
How likely are you to continue using LLMs in your future medical practice?		
	Unlikely or very unlikely	5 (7.1%)
	Neutral	9 (12.9%)
	Likely or very likely	55 (80.0%)
When utilizing LLMs, how often do you cross-check the information you receive?		
	Never	13 (18.6%)
	Occasionally	12 (17.1%)
	Sometimes	15 (21.4%)
	Often	18 (25.7%)
	Always	9 (12.9%)
	N/A	3 (4.3%)

**Figure 2:**
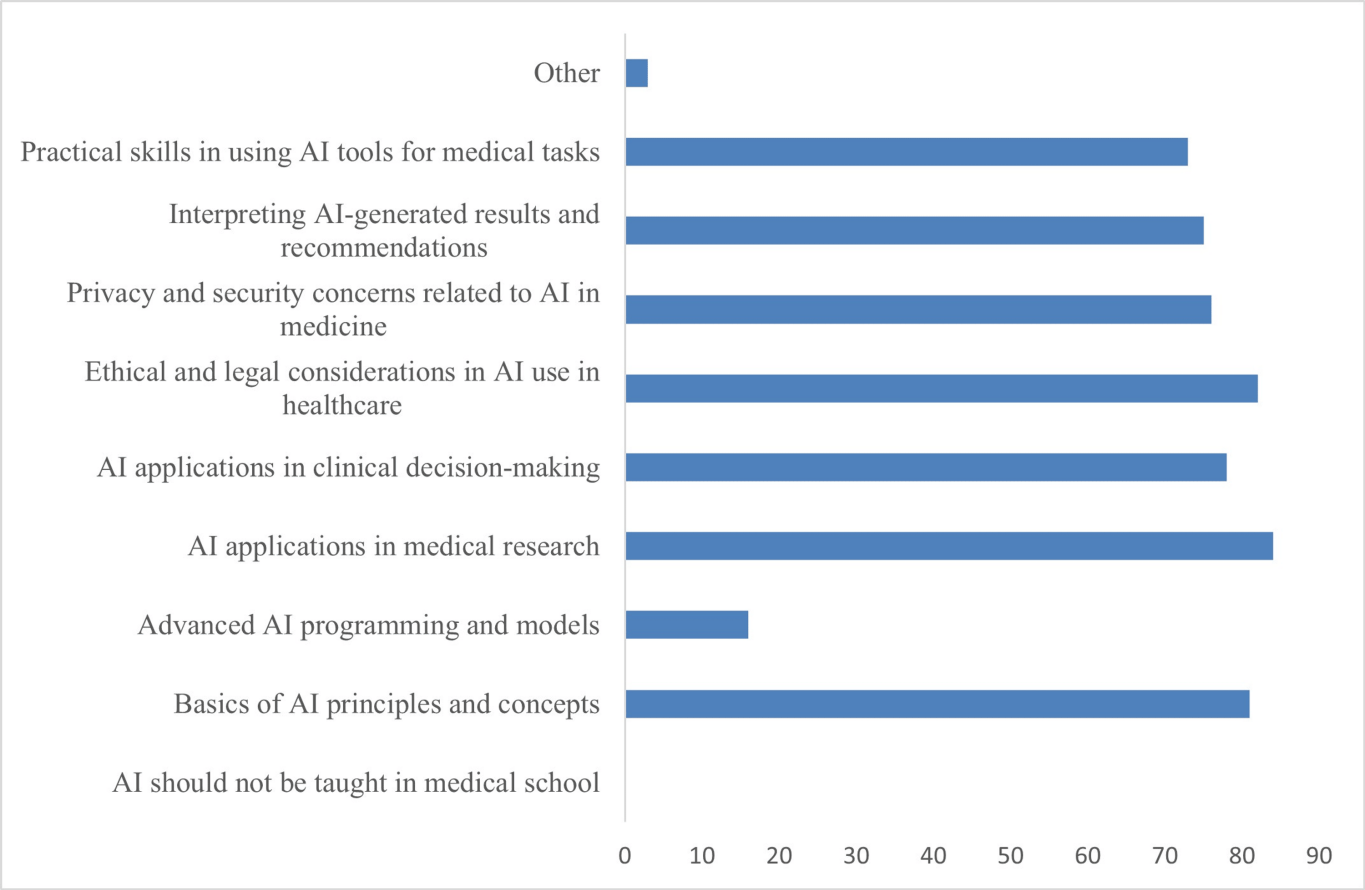
Medical students' perceptions of which aspects of AI should be taught in medical school The x-axis indicates the total number of respondents.

We also asked the entire cohort their opinions on AI in general. Thirty-three point three percent (33.3%; 34/102) of respondents reported trusting AI to assist in clinical decision-making now. Sixty point eight percent (60.8%; 62/102) responded that they were likely to trust AI to assist in clinical decision-making in the next five years (Table 3). Participants were also asked which aspects of AI should be taught in medical school; all participants believed some aspects of AI should be taught in medical school (Figure 2).

**Table 3:** Medical students' perceptions of trusting AI to assist with clinical decision-making currently and in the future n = number. Data have been represented as n, %.

Question		Medical students, n (%)
Right now, how much do you trust artificial intelligence (AI) to assist in clinical decision-making?		
	Not at all	7 (6.9%)
	Not very much	39 (38.2%)
	Neutral	22 (21.6%)
	Somewhat	32 (31.4%)
	Very much	2 (1.9%)
In the next 5 years, how much do you anticipate trusting AI to assist in your clinical decision-making?		
	Not at all	2 (1.9%)
	Not very much	16 (15.7%)
	Neutral	22 (21.6%)
	Somewhat	48 (47.1%)
	Very much	14 (13.7%)

We analyzed respondent demographics and the likelihood that they were to use an LLM, frequently cross-check information from LLMs, and trust AI with clinical decision-making in the next five years. Those with some baseline understanding of and exposure to AI were 3.26 (p=0.020) and 4.30 (p=0.002) times more likely to have used an LLM, respectively, and 5.06 (p=0.021) and 3.38 (p=0.039) times more likely to cross-check information from LLM, respectively, compared to those with little to no baseline understanding or exposure (Table 4). Furthermore, those with some exposure to AI in medical school were 2.70 (p=0.039) and 4.61 (p=0.0004) times more likely to trust AI with clinical decision-making, respectively, currently and in the next five years than those with little to no exposure (Table 4). Those who had used an LLM were 4.31 (p=0.019) times more likely to trust AI with clinical decision-making currently compared to those who had not used one (Table 4).

**Table 4:** Statistical analyses of participant characteristics and LLM use and AI perceptions OR = odds ratio, CI = confidence interval Data have been presented as OR, 95% CI, and p-value. p<0.05 is considered statistically significant. * indicates statistically significant (p<0.05) LLM: large language model

	Used LLM (OR, (95% CI), p-value)	Frequently cross-checks information from LLMs (OR, (95% CI), p-value)	Trust AI with clinical decision-making currently (OR, (95% CI), p-value)	Trust AI with clinical decision-making in the next 5 years (OR, (95% CI), p-value)
Clinical phase (3rd or 4th year)	0.40 (0.15-1.08, p=0.064)	2.60 (0.65-10.43, p=0.167)	0.41 (0.14-1.22, p=0.104)	0.50 (0.20-1.24, p=0.132)
Pursuing procedural specialty	2.10 (0.69-6.39, p=0.183)	1.27 (0.41-3.92, p=0.682)	0.93 (0.37-2.34, p=0.875)	0.62 (0.26-1.51, p=0.295)
Some current understanding of AI	3.26 (1.17-9.12, p=0.020)*	5.06 (1.17-21.84, p=0.021)*	1.09 (0.40-3.00, p=0.865)	1.39 (0.54-3.60, p=0.499)
Some exposure to AI in medical school	4.30 (1.62-11.39, p=0.002)*	3.38 (1.03-11.08, p=0.039)*	2.70 (1.03-7.06, p=0.039)*	4.61 (1.92-11.02, p=0.0004)*
Used LLM	-	-	4.31 (1.18-15.71, p=0.019)*	2.50 (0.97-6.45, p=0.0534)

## Discussion

Overall, medical students in our survey were both aware of LLMs and actively incorporating them as an educational tool. Respondents' opinions of LLMs were generally positive in terms of perceived accuracy and likelihood of future use. Moreover, participant perceptions of AI were also positive, with all believing AI should be incorporated into medical education.

Unsurprisingly, our study indicated medical students with prior baseline knowledge of or exposure to AI in medical school were more likely to have used LLMs. Those who reported some exposure to AI in medical school were also more likely to report trust in AI to assist with clinical decision-making in the next five years. Our finding was consistent with results from a similar cross-sectional study performed at an international academic institution, which found that medical students’ previous experience with AI was significantly associated with positive AI perceptions such as improving patient care, decreasing medical errors and misdiagnoses, and increasing the accuracy of diagnoses [8]. Importantly, we were also interested in how likely medical students were to engage in conscientious AI use habits such as cross-checking information obtained from LLMs. In our current survey, we did not specifically ask or define what resources students use to cross-check information; it was used as a metric of awareness of the need to cross-check information when using many currently available LLMs. Current LLMs and LLM-powered chatbots like ChatGPT are not infallible sources, and there is a well-known tendency to fabricate data, or “hallucinate”, in a manner where the generated responses appear convincing and correct, which can lead to medical error if users do not cross-check [3,12-15]. Additionally, LLM response quality and accuracy are also affected by users’ search prompts. The ability to form a prompt with appropriate clinical information will likely vary based on educational level, clinical exposure, and knowledge of LLMs. Insufficient training and knowledge of AI limitations risk overreliance, which could lead to gaps and inaccuracies in education or ultimately medical error. We found that medical students with some prior understanding of AI or exposure to AI in medical school were more likely to engage in conscientious AI use habits such as frequently cross-checking information.

AI tools, such as LLMs, can also easily be integrated into early medical pre-clinical education. Standardized testing remains a metric for medical student evaluation and while many paid third-party medical information and question bank resources already exist, LLMs offer a free platform for medical students to generate practice questions in preparation for shelf and standardized exams. The dynamic, conversational nature of many LLMs also makes for an excellent facilitator of small (peer) group education, which includes problem-solving and working through various clinical case presentations; small-group education has been shown to be a highly effective teaching method [16,17]. Other proposed benefits include receiving input from student users to develop tailored study plans and schedules or drafting research papers [18]. Students are not the only LLM users, as they can be a practical tool for medical educators creating clinical vignettes for exam questions or assessment plans and rubrics for students [18]. Indeed, according to recent survey results on medical education faculty, many instructors have already begun using applications such as ChatGPT to create multiple-choice questions [5]. LLMs can further be incorporated into medical education as fictitious or even standardized patients to help students practice taking patient histories and developing an assessment and plan [19]. Further down the line, LLMs may also be of educational benefit to practicing physicians in creating medical cases in preparation for board exams.

LLMs also pose unprecedented challenges in medical education. The ability of LLMs to draft essays and complete written assignments raises issues with academic dishonesty and plagiarism [3]. While several tools have been developed to detect AI-generated text, one study has found that the addition of one word to AI-written text can reduce detection from 99% to 24% [20]. The accuracy and reliability of information obtained from current LLMs also vary for several reasons related to both the user and the algorithms. The concept of hallucination was brought up previously, but other issues may arise with accuracy including LLMs not being trained on the most up-to-date data and algorithmic bias by pulling information from unrepresentative sources of information. Additionally, LLM responses also vary significantly based on user prompts leading to significant variability in educational value. While the performance of LLMs is certainly expected to improve over the years, another concern for medical trainees and professionals down the line is the risk of overreliance on LLMs. Caution must be taught and exercised in using AI to supplement, not supplant clinical reasoning and decision-making.

While we have learned that LLMs are actively being used by medical students, the next steps are to examine the specific use cases within the categories we initially explored (i.e. academic studies, patient or clinical-related tasks, research) in our future national study. For example, we would like to elucidate if those who were using LLMs for clinical care were using them specifically to write medical notes, generate differential diagnoses for a patient presentation, etc. We also plan to amend the response options to make the data more conducive for 2x2 statistical analysis, as the “neutral” response option introduced ambiguity in the interpretation of the data. For example, 10% (n=7) of participants responded “neutral” when asked to rate the accuracy of responses provided by LLMs. It was not clear whether this indicated true uncertainty about the accuracy of LLM responses or if it reflected an equal proportion of accurate and inaccurate responses provided by LLMs. In the next study, we plan to remove the “neutral” option and clarify in the response options if LLMs were giving accurate responses more often than inaccurate responses. Moreover, in addition to Likert scale responses, we find it would be helpful to give quantitative response options, such as providing the relative percentage of correct information that LLMs provide, as an example.

The adoption of an AI curriculum in medical school bodes well for the future likelihood of AI adoption in the medical field as these technologies inevitably improve. Our preliminary findings support the need for education on and integration of AI tools into the medical curriculum, not just to prepare future physicians to use advanced technologies in the future but also to encourage better use habits and more avid acceptance of AI technology such as LLM-based chatbots. All surveyed participants believed that some aspects of AI should be taught in medical school. This highlights a key need to adapt the medical curriculum to the unique benefits and challenges that AI will bring to the medical field. Undergraduate and graduate medical education training will be a key driving force for exposing future physicians to AI early and encouraging effective use of it.

Our study is limited in several ways. First, it is a pilot survey with a small sample size at a single institution. In addition to the small sample size, there is also a limited representation of medical students in their third and fourth years of clinical training, who likely possess a better understanding of clinical patient care and a stronger foundation of medical knowledge. As it was a pilot study performed at a single tertiary care academic institution with an AI elective in pre-clinical years, it may not be generalizable to institutions without AI instruction. All participants had heard of LLM applications, such as ChatGPT, indicating there is likely a selection bias. In the future, we aim to survey medical students from a broader range of medical institutions across the United States, as well as to inquire about the perceptions of more advanced medical trainees such as residents and attending physicians.

## Conclusions

Popular AI tools, such as LLMs, are already making their way into the medical student repertoire of study resources. Medical students who participated in the survey generally had a positive perception of LLMs and reported they were likely to continue using them in the future. All respondents believed AI should be taught in the medical curriculum, highlighting a key opportunity in medical education to acclimate medical students to AI.
